# Design and evaluation of an open-source, conformable skin-cooling system for body magnetic resonance guided focused ultrasound treatments

**DOI:** 10.1080/02656736.2021.1914872

**Published:** 2021

**Authors:** Robb Merrill, Henrik Odéen, Christopher Dillon, Rachelle Bitton, Pejman Ghanouni, Allison Payne

**Affiliations:** a Department of Radiology and Imaging Sciences, University of Utah, Salt Lake City, UT, USA; b Sandia National Laboratories, Albuquerque, NM, USA; c Department of Radiology, Stanford University, Stanford, CA, USA

**Keywords:** MRgFUS, HIFU, skin burns, focused ultrasound, skin cooling

## Abstract

**Purpose:**

Magnetic resonance guided focused ultrasound (MRgFUS) treatment of tumors uses inter-sonication delays to allow heat to dissipate from the skin and other near-field tissues. Despite inter-sonication delays, treatment of tumors close to the skin risks skin burns. This work has designed and evaluated an open-source, conformable, skin-cooling system for body MRgFUS treatments to reduce skin burns and enable ablation closer to the skin.

**Methods:**

A MR-compatible skin cooling system is described that features a conformable skin-cooling pad assembly with feedback control allowing continuous flow and pressure maintenance during the procedure. System performance was evaluated with hydrophone, phantom and *in vivo* porcine studies. Sonications were performed 10 and 5 mm from the skin surface under both control and forced convective skin-cooling conditions. 3D MR temperature imaging was acquired in real time and the accumulated thermal dose volume was measured. Gross analysis of the skin post-sonication was further performed. Device conformability was demonstrated at several body locations.

**Results:**

Hydrophone studies demonstrated no beam aberration, but a 5–12% reduction of the peak pressure due to the presence of the skin-cooling pad assembly in the acoustic near field. Phantom evaluation demonstrated there is no MR temperature imaging precision reduction or any other artifacts present due to the coolant flow during MRgFUS sonication. The porcine studies demonstrated skin burns were reduced in size or eliminated when compared to the control condition.

**Conclusion:**

An open-source design of an MRgFUS active skin cooling system demonstrates device conformability with a reduction of skin burns while ablating superficial tissues.

## Introduction

Magnetic resonance-guided focused ultrasound (MRgFUS) has emerged as a promising therapeutic technology to displace traditional therapies for the control and elimination of soft tissue tumors, including uterine fibroids [1,2], desmoid tumors [3–5], and vascular malformations and pancreatic cancer [6,7]. The application of MRgFUS to the treatment of desmoid tumors is particularly important, as these tumors are locally aggressive tumors that can infiltrate healthy tissues at many body sites, causing pain and functional impairment [8]. Additionally, desmoid tumors often recur after surgery, and respond incompletely to radiation and medical therapies, which also have significant adverse side effects [9]. While focused ultrasound has successfully treated these tumors, the most common complications are skin burns, especially when treating large tumors, superficial tumors, or tumors near surgical scars.

All feasibility studies applying focused ultrasound to desmoid tumors have reported unintended first, second, and even third-degree skin burns [3–5,10]. The largest study noted skin burns in eight out of fifteen patients, with second-degree burns often occurring if the target tissue was superficial (*n* = 6, average 4 mm from skin) [4]. Focusing the beam at superficial targets introduces relatively high ultrasound intensities at the skin that can lead to burns.

MRgFUS trials and clinical protocols typically use tumor-to-skin proximity less than 1.0 cm as an exclusion criterion with the intention of avoiding skin burns [3,11,12], thereby reducing the number of patients eligible for this noninvasive therapy. If the patient is not excluded outright, the superficial portions of the tumor may not be targeted during treatment, reducing the likelihood of achieving an enduring clinical benefit.

Even when the target is deep, the numerous sonications (routinely numbering more than a hundred) required to fully ablate a large desmoid tumor will repeatedly expose the skin to low-intensity ultrasound for extended periods of time [13,14], which can cumulatively also lead to skin damage, even third-degree burns [5]. Absorption of heat is particularly significant around surgical scars. Identifying skin burns before they occur is particularly challenging both because MR thermometry is unreliable in skin and subcutaneous fat and because desmoid tumor patients are under general anesthesia, making skin in the ultrasound pathway not immediately accessible for visual monitoring. Importantly, the risk of skin burns when treating either superficial or deep tissues is not limited to desmoids and remains a major limiting factor in treating any target with MRgFUS [2,15–21].

MRgFUS devices utilize an inter-sonication delay period between successive sonications in an attempt to mitigate the risk of skin-burns [22]. For large tumors that may require in excess of one hundred sonications, this cooling requirement can extend the total treatment time by multiple hours [23]. Indeed, average total treatment time for the multicenter desmoid tumor study was 3.5 h with one treatment lasting 8h [4]. Some applications of MRgFUS, such as uterine fibroids, are restrained to 3-h treatment sessions [24], which, considering large tumor volumes, may require the patient to return for additional treatment sessions or limit the targeted volume within the tumor. Even for tumors located deeper in the body, the use of active cooling would allow reduction in treatment time by reducing the required inter-sonication time to maintain treatment safety.

For desmoid tumors, extended treatments increase the time patients are sedated under general anesthesia, which, especially for younger patients, should be minimized [25]. Other MRgFUS applications, such as the treatment of uterine fibroids, employ conscious sedation. These patients may move during treatment, due to discomfort from heating of the skin and from prolonged prone positioning. Patients may also become more uncomfortable, restless, or claustrophobic in the confining MRI bore as total treatment time increases. Any movement requires alterations to the treatment plan, further increasing treatment time. These extensions increase costs, making long treatment times another major limiting factor of MRgFUS therapies [23,26]. Strategies for improving time-efficiency without sacrificing safety or efficacy have great potential for improving the feasibility, attractiveness, and cost-efficiency of MRgFUS treatments [26–30].

Some focused ultrasound systems implement active skin cooling during treatments. The Insightec Exablate Neuro system flows water over the large transcranial transducer between sonications, circulating chilled water around the head to cool the cranium and scalp [31]. The Insightec conformable bone system has a water-permeable membrane to provide effective acoustic coupling and integrated built-in skin cooling [32]. Additional skin cooling devices include the Profound Sonalleve MRgFUS direct skin cooling (DISK] device for the treatment of uterine fibroids [30]. The platform-specific DISK device includes a rigid horizontal channel upon which the patient lays and through which room temperature water flows to increase convective cooling of the skin. During sonications, the DISK device is turned off to avoid flow artifacts in the associated MR monitoring images and ultrasound passes through two mylar membranes (the upper and lower surfaces of the water-filled channel) and then into the patient’s body. After the MR thermometry monitoring has concluded, the DISK device is turned on to remove excess thermal energy deposited in the skin. This active cooling reduces energy accumulating in the skin and subcutaneous fat layers to prevent skin burns and is also used to guide a treatment control platform that reduces cooling times and, by extension, overall treatment times. The device is only compatible with the Sonalleve MRgFUS system, and its design works well for coupling ultrasound to the broad abdomen in uterine fibroid treatments. However, the non-conformable horizontal acoustic window would not work effectively for treating body sites with greater curvature, such as in the extremities where most desmoids occur. LIPOcel is a focused ultrasound device for subcutaneous fat reduction and body contouring that also employs active skin cooling; however, it is not MRI-compatible [33]. Others have used convective cooling with water or ice baths to limit the development of skin burns [5,13,34]. However, low temperature coolants have also caused hypothermic skin damage. While other medical applications utilize skin cooling devices, those devices have not been designed for ultrasound transparency and generally are not MRI compatible [35–37].

The objective of this design-driven study is to provide a validated, conformable, forced convective skin-cooling device that can be integrated with existing body MRgFUS systems to more effectively prevent skin burns and reduce lengthy treatment times. The system design is presented as an open source resource with all specifications required for the duplication of the skin-cooling device. System evaluation is presented with (i) bench hydrophone measurements, (ii) non-heating human, and (iii) heating phantom studies, as well as an (iv) ablation study in a porcine model under both skin cooling and control conditions.

## Materials & methods

An MR-compatible skin-cooling system has been designed and constructed for body MRgFUS treatments. Critical design requirements included MR compatibility, acoustic transparency, conformable acoustic coupling capability and maintenance of skin temperature for skin burn prevention. Additional requirements were patient weight support while maintaining consistent pressure, air bubble prevention and patient comfort.

The skin cooling system is a forced convective cooling device with user-specified controls that can modify both skin-cooling pad pressure and coolant temperature. Distilled, degassed water is used as the cooling fluid, eliminating potential biocompatibility issues. A comprehensive description of all components, including supplier sources, assembly instructions and user manual is located in the open source design documents (https://github.com/fuslab-uofu/SkinCoolingDevice).

**Figure 1.**
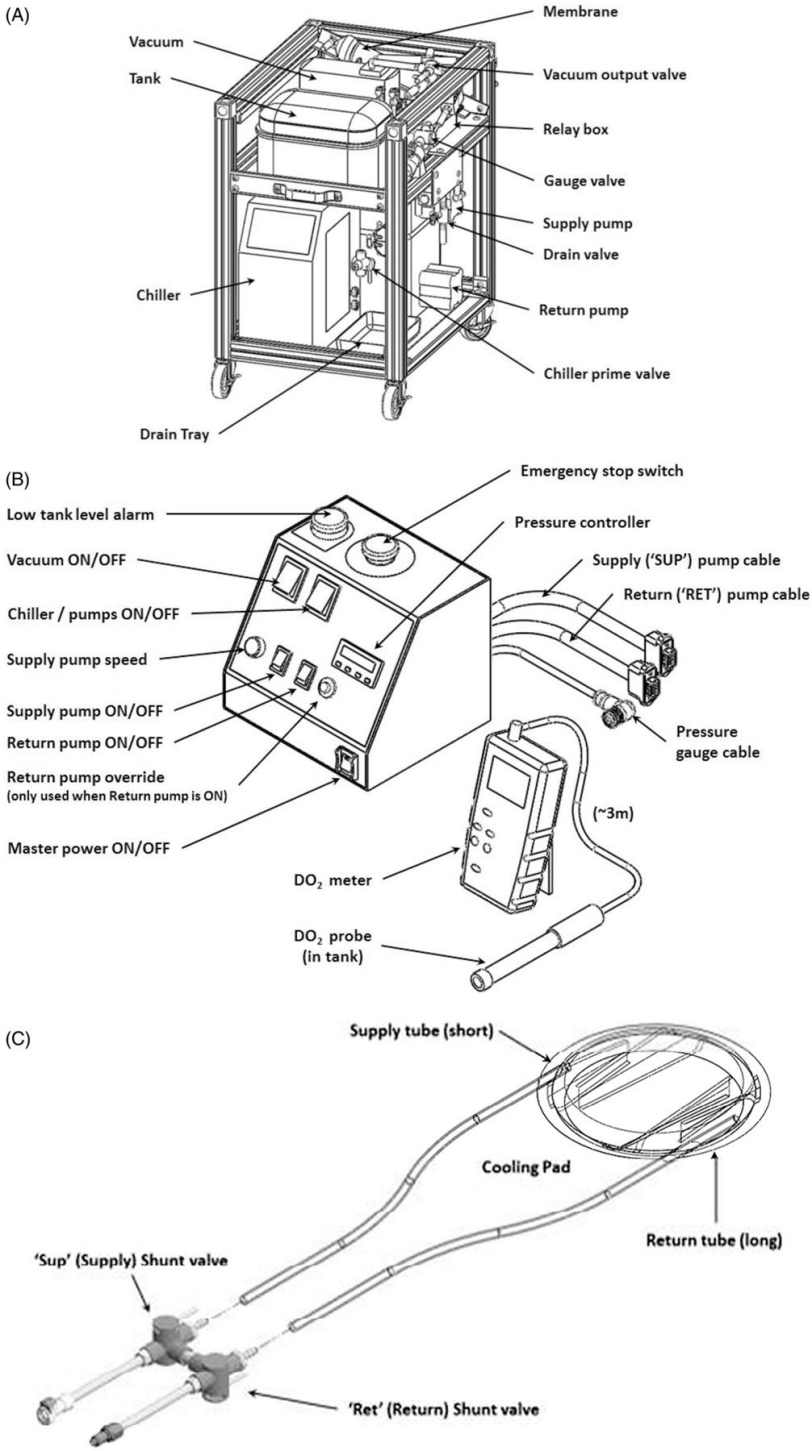
Main components of the skin cooling system. (a) Schematic of the cart components including pumps, degasser and water reservoir. The cart is designed to be placed under a countertop work space and is sited in the MR control room. (b) Schematic of the control box that contains feedback control electronics and all power switches. The control box is placed adjacent to the MRgFUS control computer for ease of access. A separate dissolved oxygen meter is placed by the control box in order to monitor the dissolved oxygen content in the water reservoir. (c) Skin-cooling pillow assembly. Insulated input and output tubes are routed into the MRI suite via the waveguide and quick connect attachments are attached to the skin-cooling pillow. The pillow should be inspected before every treatment.

The system consists of three main components: (i) a component cart that houses the chiller, supply and return pumps, a water reservoir and an inline degassing system. This cart is designed to sit outside the MRI room; (ii) a skin-cooling pad assembly that includes a welded pad with flow baffles, supply tubes and shunt valves; and (iii) a control box that is positioned adjacent to the MRgFUS control computer and contains the pressure controller, emergency stop switch, system alarms and pump power switches. Each component is shown in Figure 1 and briefly described below.

### System design

#### Component cart

**Figure 2.**
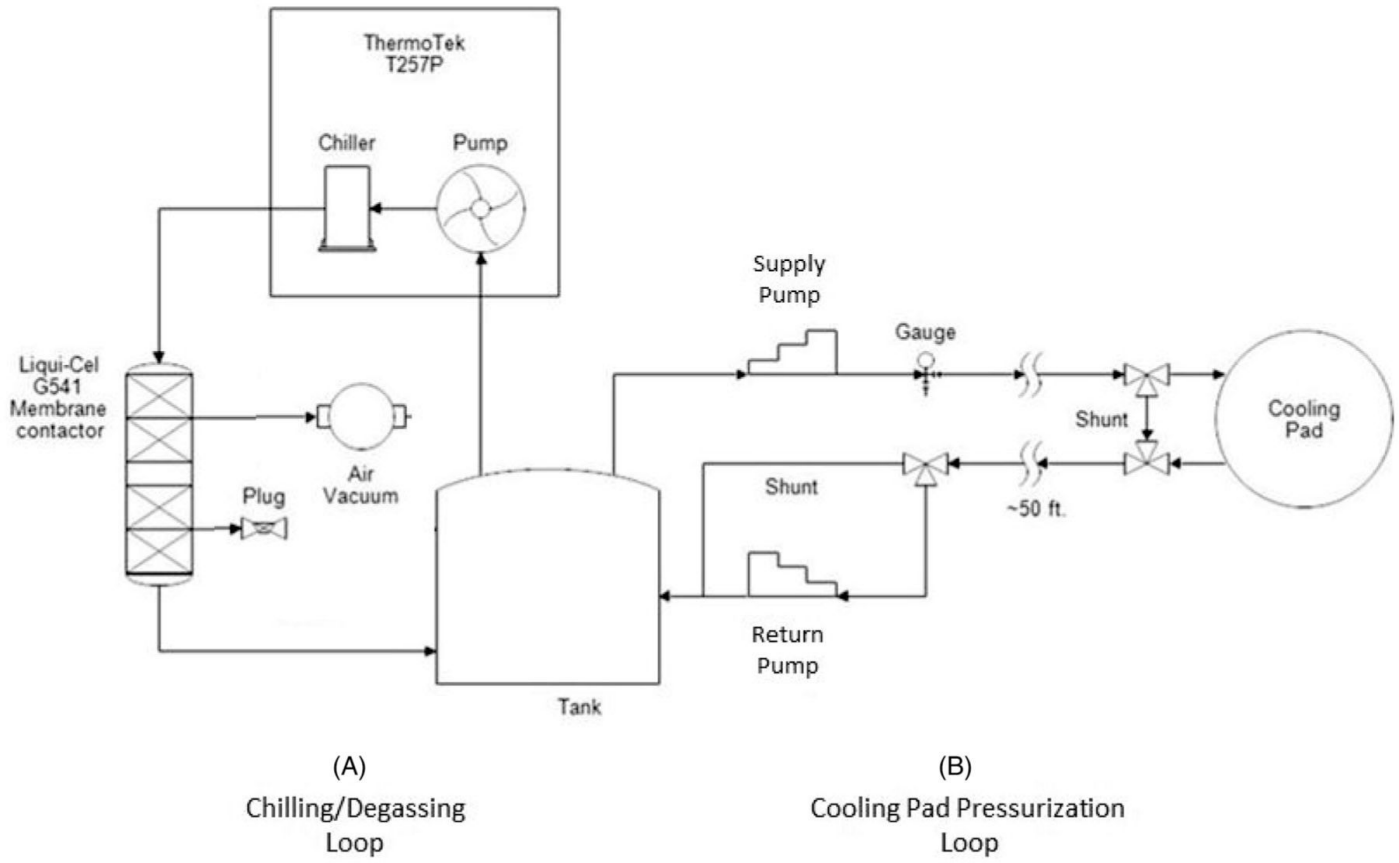
Simplified plumbing diagram indicating the role of each component located on the component cart. Loop (A) chills the coolant while loop (B) delivers the coolant to the skin cooling pad assembly.

The component cart was designed to be sited under a counter in the MRI control room. The cart contains the components seen in Figure 1(a) including a water reservoir tank, degassing system including a vacuum pump, filter, membrane and vacuum output valve. Supply and return pumps control the flow to and from the skin-cooling pad assembly. The chiller pump cools the distilled water that is used as the coolant. The controls to set the target temperature for the coolant fluid are located on this assembly. The cart consists of two plumbing circuits shown in the simplified plumbing diagram in Figure 2. In Loop (A), the chiller unit both chills and pumps the water through the degassing membrane. A vacuum unit removes dissolved oxygen from the water through this membrane, preventing bubble buildup from forming in the cooling pad. Loop (B), the cooling pad loop, has two different operating modes. Mode 1, *System startup*: Both ‘shunt’ lines (near the pad and across the return pump) are activated to circulate chilled water through the system while preventing bubbles from entering the cooling pad and reducing pad wear. Only the supply pump is active during this mode. Mode 2, *Normal operation:* The ‘shunt’ lines are deactivated, and both pumps actively maintain the cooling pad at a constant pressure. The supply pump delivers chilled water from the tank to the cooling pad at a constant flow rate. The return pump draws water from the cooling pad back to the tank at a variable rate controlled by continual feedback from the pressure gauge, using a proportional-integral-derivative (PID) temperature controller repurposed for pressure control.

#### Control box

A custom control box, shown in Figure 1(b), contains both a potentiometer and a PID pressure controller to alter the flow rates of the supply and return pumps, respectively, to maintain a constant pressure in the skin-cooling pad. The control box also contains the power switches to all pumps, as well as On/Off switch for in-line vacuum pump used to degas the water, a low coolant level alarm, and an emergency stop button. The control box also supplies power to the relay and solenoid shunt valve that bypasses the return pump for chilling and bubble removal during startup mode. A separate dissolved oxygen meter is placed adjacent to the control box in order to monitor the dissolved oxygen content in the water reservoir on the component cart.

The pressure controller consists of a repurposed Temperature Controller (AutomationDirect SOLO SL4824-LR), a digital pressure sensor (ProSense), and two variable speed peristaltic pumps (MasterFlex) for supply and return flow. The speed of the supply pump is fixed at the desired input flow rate for the cooling pad (0.5 L/min for this study). The controller features a 150 ms sample time from the analog pressure sensor signal, which results in a rapid response time for proportional-integral-derivative (PID) control of the return pump speed to regulate cooling pad pressure during normal operation. A separate controller alarm function drives the pump at maximum speed in case of pad over pressurization. The System User Manual, provided in the open source design documentation, describes how to adjust the pressure settings on the controller for considerations such as barometric pressure, anatomy type, and conformability.

#### Skin-cooling pad assembly

The skin-cooling pad assembly is made of welded polyether film material of 0.15 mm thickness, as seen schematically in Figure 1(c). A roller welder was used to create the seals around the edges and to define the baffled channels, constructed using the same polyether film material. Channels help restrict pad thickness when pressurized, and are only located on the edges of the pillow to provide a 10 cm window for ultrasound beam propagation through the center of the pad. Tubing ports were glued into openings at opposite sides of the pad for input/output coolant flow. Construction details of the skin-cooling pad construction can be found in the open-source documentation. The skin-cooling pad can be disinfected between treatments, but it is designed to be a disposable device. The usable life of the pad assembly is a function of weight placed on the pad and duration of treatment. Visual inspection of the weld quality, as described in the system user manual, can be used to determine pad usability. Approximately 12 meters of dual-channel insulated tubing conveys chilled coolant from the cart in the MRI control room, through the MRI waveguide to the cooling pad within the MRI scanner. The coolant flow rate was set to 0.5 L/min for this study, with a working pressure of 125 mbar (at 1300 meters elevation). Coolant temperature was maintained at 5–10 °C. The size of the skin-cooling pad is approximately 22 cm in diameter when inflated. However, the diameter of the pad can be potentially customized to specific indications.

### Safety measures

Several safety measures are incorporated into the presented skin-cooling system. First, a ‘Low Tank Level’ sensor alerts the user if the amount of water coolant in the tank falls below approximately 3 liters. This alarm helps prevent bubbles from entering the system and the cooling pad, which would end the treatment session immediately, and helps alert the operator if the cooling pad has developed a leak. Second, the electronic pressure controller is programmed to rapidly remove coolant from the cooling pad (at the maximum flow rate of the peristaltic pump) if the pressure rises above a specified amount (set pressure + 15 mbar by default, adjustable by the operator). This function serves as an ‘over-pressure relief valve’ for the system if an emergency such as a kinked line should develop, or during normal operation while the pressure oscillations settle after the system pumps are turned on. Third, the skin-cooling pads are ideally only used for a single treatment, since irreversible wear-and-tear can occur during treatment. Should cooling pads be used multiple times without careful inspection, it is the operator’s responsibility to ensure that the MR table and any other water-sensitive components are adequately protected from the potential of 3–4 liters of water pooling in the table. More detailed operating instructions are provided in the System User Manual provided in the open-source design documentation.

### System evaluation

All MRgFUS studies were performed with a preclinical MRgFUS system (*f* = 950 kHz, 256-element phased-array transducer, 13 cm radius of curvature, 1.8 × 1.8 × 8 mm full width half maximum pressure pattern in water, Image Guided Therapy, Inc., Pessac, France) in a 3 T MRI scanner (Siemens Prisma^FIT^, Erlangen, Germany). Human studies were conducted under local Internal Review Board approval and all animal studies were approved by the Institutional Animal Care and Use Committee.

#### Bench hydrophone testing

**Figure 3.**
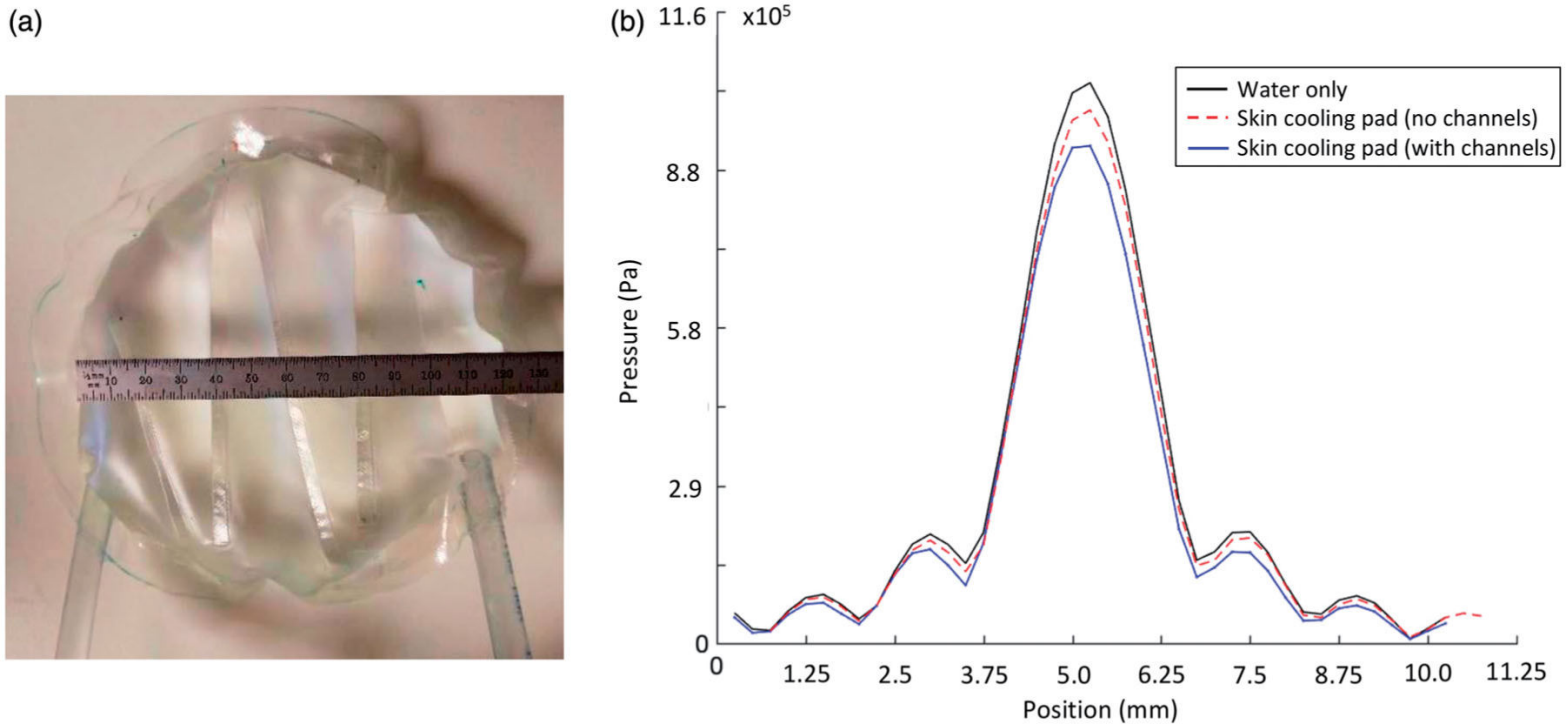
Skin cooling pillow configuration used for hydrophone tests. (a) Small-scale skin-cooling pillow (with channels) placed in the near field of the focused ultrasound beam for hydrophone testing. (b) Transverse profile of the pressure measured under water only and skin-cooling pillow conditions with and without channels. No beam profile distortion occurs with the skin-cooling pillow in place. Pressure decreases 5–12% depending on the presence of channels in the near-field of the ultrasound beam.

Acoustic transparency of the skin-cooling pad assembly was evaluated using scanning hydrophone measurements. Two small-scale (12 cm diameter) testing pads (Figure 3(a)) were placed in the near field of a focused ultrasound transducer (256 semi-randomly positioned elements of 4 mm diameter, *f* = 940 kHz, 14.4 × 9.8 cm aperture, 10 cm radius of curvature) mounted vertically in a degassed water bath with a hydrophone (HNR-500, Onda Corporation, Sunnyvale, CA, USA) scanned with two stepper motors (NRT150, Thorlabs Inc., Newton NJ, USA) in the plane of the transducer’s geometric focus. Electronics for driving the transducer were designed and constructed by Image Guided Therapy, Inc. (Pessac, France). Two-dimensional pressure patterns were obtained over a 1 × 1 cm grid with hydrophone scans (0.25 mm isotropic spacing) with both the small-scale testing pillows in place and water only conditions. The testing pads mimicked the skin-cooling pad both with and without channels in place. The 2D pressure patterns were propagated to 3D using an angular spectrum propagation technique [38].

#### Non-heating human study

Skin-cooling pad assembly conformability to different anatomies was evaluated in a healthy human volunteer. The pad was placed under various anatomies to demonstrate how placement and acoustic coupling could be achieved in the targeting of different targets. Positioning and coupling was evaluated using a 3D T1-weighted VIBE sequence (TR/TE = 5.89/1.89 ms, 1.3 × 1.3 × 2 mm resolution, FA = 10°, 250 Hz/pixel bandwidth).

#### MRgFUS phantom study

**Figure 4.**
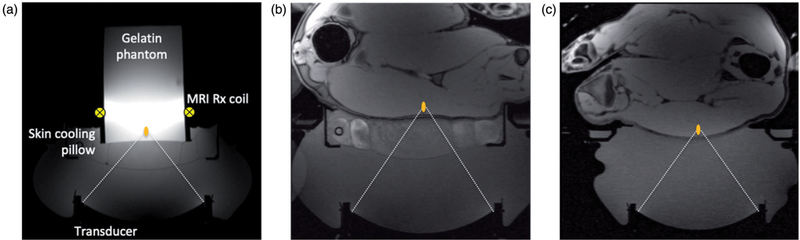
Experimental setup for the MRgFUS studies. In all cases a schematic focus is shown 1 cm from the subject interface. (a) Gelatin phantom placed on the skin-cooling pillow with the placement of the single loop MRI receive coil indicated schematically. Axial T1-weighted images of porcine subject (b) with hind leg coupled to the skin-cooling pad assembly and (c) in the control setup where no skin-cooling pad was utilized.

A phantom study was performed to quantify the effects of the skin-cooling pillow on MR thermometry precision. A 250-bloom gelatin phantom was constructed as has been previously described [39]. The phantom was placed on the skin-cooling pillow, as seen in Figure 4(a), with a single loop radiofrequency MRI receive coil placed around the phantom. The transducer, skin-cooling pillow and phantom were acoustically coupled with degassed, deionized water. Single MRgFUS sonications (20 W acoustic, 20.75 s) were focused 1 cm from the bottom of the phantom. Separate sonications were applied with MR temperature images acquired in the sagittal (phase encode direction head/foot), coronal (phase encode direction right/left) and axial (phase encode direction right/left) orientations (2 D gradient echo sequence with segmented EPI readout, ETL = 11, TR/TE = 247/13 ms, FA = 41°, 1.9 × 1.9 × 3.6 mm resolution, 1775 Hz/pixel bandwidth). Both flow on and flow off conditions were evaluated. The flow rate was set at 0.5 L/min with a target coolant temperature of 5 °C. Flow was continuous during the flow on condition. Field drift correction was performed by selecting a 1 cm^3^ non-heated volume adjacent to the heated region, obtaining the mean of that non-heated region and subtracting the non-heated mean field drift from all temperature images through time. Temperature measurement precision was further quantified by computing the spatial mean of the standard deviation through time of temperature measurement over a 1 cm^3^ non-heated region (different from drift correction region) in the phantom.

#### Porcine model MRgFUS ablation study

**Figure 5.**
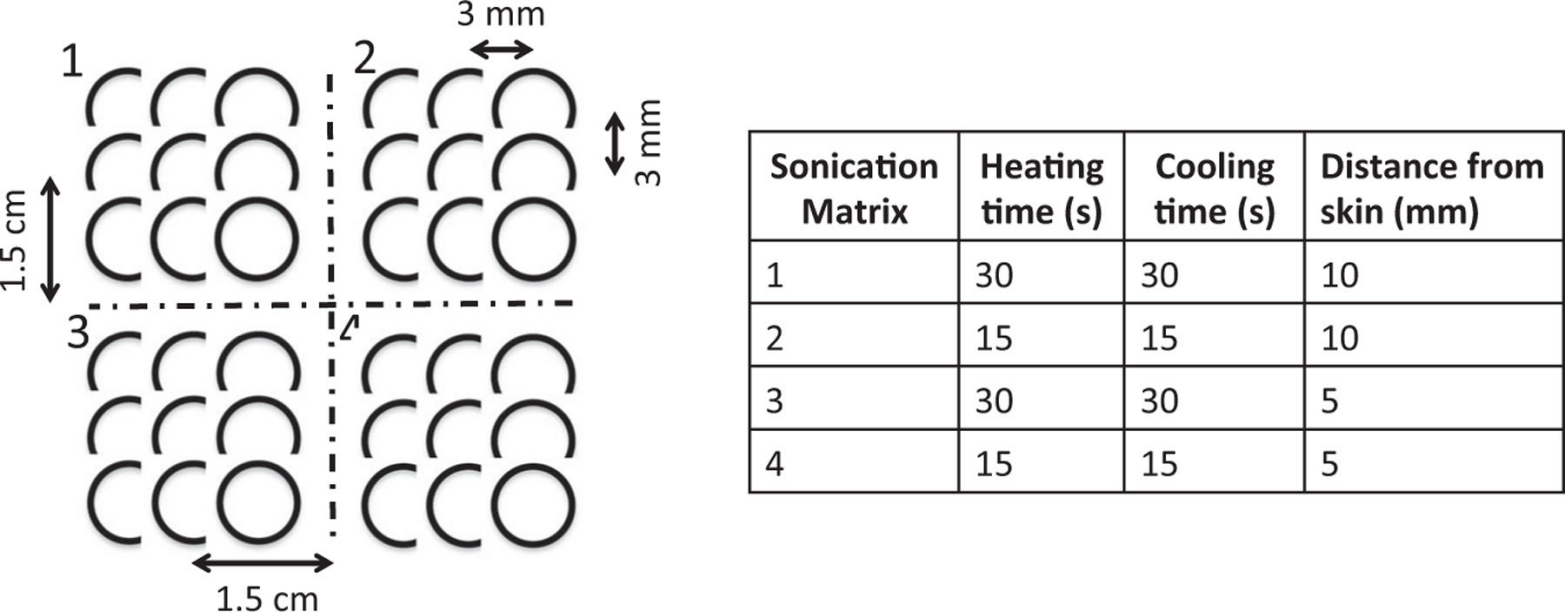
Sonication matrix parameters applied to both the control and skin-cooling ablation sides in the porcine model. In all cases, the power for individual sonications were adjusted to achieve a 65 °C peak temperature, independent of the heating or cooling times.

*In vivo* effects of the skin cooling system were evaluated in a porcine model. MRgFUS ablations were performed in the haunches of three farm pigs (~30 kg, Premier BioSource, Ramona CA). Animals were anesthetized with an intramuscular injection of telazol, ketamine and xylazine (4.4, 2.2 and 2.2 mg/kg, respectively). The animal was then intubated and anesthesia was maintained with inhaled isoflurane (1–4%). The haunch of the animal was shaved and a depilatory cream was applied (Nair, Church & Dwight, Ewing, NJ). The animal was placed on top of the MRgFUS system and two Siemens 4-channel multi-purpose flex coils were placed over the animal. Animals were monitored continuously throughout the treatment with end tidal CO_2_ capnography, pulse oximetry and rectal temperature. Four sonication matrices were ablated in a 2 × 2 grid pattern. Each sonication matrix consisted of nine single point sonications configured in a 3 × 3 pattern. The focal plane of sonication matrices 1 and 2 were placed 10 mm from the skin while sonication matrices 3 and 4 were placed 5 mm from the skin. The sonication patterns and heating and cooling schemes for each of the matrices are indicated in Figure 5. The applied power was calibrated for each animal for each treatment condition (control and skin cooling) to achieve a peak temperature of approximately 65 °C. The sonications were all performed under control conditions (no skin-cooling pad assembly) on one side of the animal, the animal was then flipped and the sonications were repeated with the skin-cooling pad assembly in place. In all cases the flow rate was set at 0.5 L/min with a coolant temperature of 5–10 °C. The mean time between completing the control and skin-cooling condition ablations was approximately 3 h. The experimental setup for both the skin cooling and control conditions is seen in Figures 4(b,c). MRTI was monitored in real time using a 3D gradient echo sequence with a segmented echo planar imaging readout (coronal orientation, TR/TE = 25/14 ms, ETL = 7, FA = 13°, resolution 2 × 2×2 mm, bandwidth = 1002 Hz/pixel, acquisition time = 4.5 s). Cumulative thermal dose for each sonication was computed following Sapareto and Dewey [40]. The base temperature for these calculations was assumed to be the animal’s body temperature, which was monitored throughout the study with a rectal fiberoptic temperature probe. At the end of the study, the animal was euthanized and a gross analysis of the skin and any resulting skin burns was performed.

## Results

The presence of the skin-cooling pad assembly in the near field of the ultrasound beam path does not cause any beam distortion or positional change; however, as seen in Figure 3(b), a 5 to 12% reduction of peak pressure was observed when comparing to the water only condition. There was no deviation in beam size due to the presence of the skin cooling pad assembly, with the full-width-half-maximum measurements of the pressure pattern being 1.77 × 10.24 mm and 1.76 × 9.74 mm for the skin-cooling and control cases, respectively.

**Figure 6.**
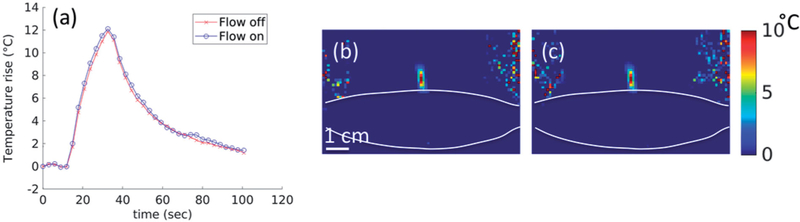
Effect of flow on MR temperature imaging. Results from sagittal MRTI acquisition in a gelatin phantom acquired during flow off and flow on conditions. (a) Peak temperature achieved in the phantom during both flow conditions. 2D temperature image in the sagittal plane and shown for the (b) flow off and (c) flow on conditions. The skin-cooling pillow placement is indicated by the white lines. White scale bar in (b) is 1 cm with an equivalent scale in (c).

The skin-cooling pad in an active flow condition did not cause any artifact or additional noise in the MR temperature imaging. Figure 6 shows the phantom results for MR temperature images acquired during flow off and flow on conditions. The temperature rise corrected for field-drift (Figure 6(a)) showed no measurable difference between peak temperatures during flow off and on conditions. Similar results are seen in the 2D spatial peak temperature maps shown in Figure 6(b,c). The temperature precision in the flow off and on conditions was 0.048 and 0.054 °C, respectively. There was no impact on image quality as well, with relative SNR values of 304.1 and 299.7 measured 1 cm from the skin-cooling pad interface during the flow off and on conditions.

**Figure 7.**
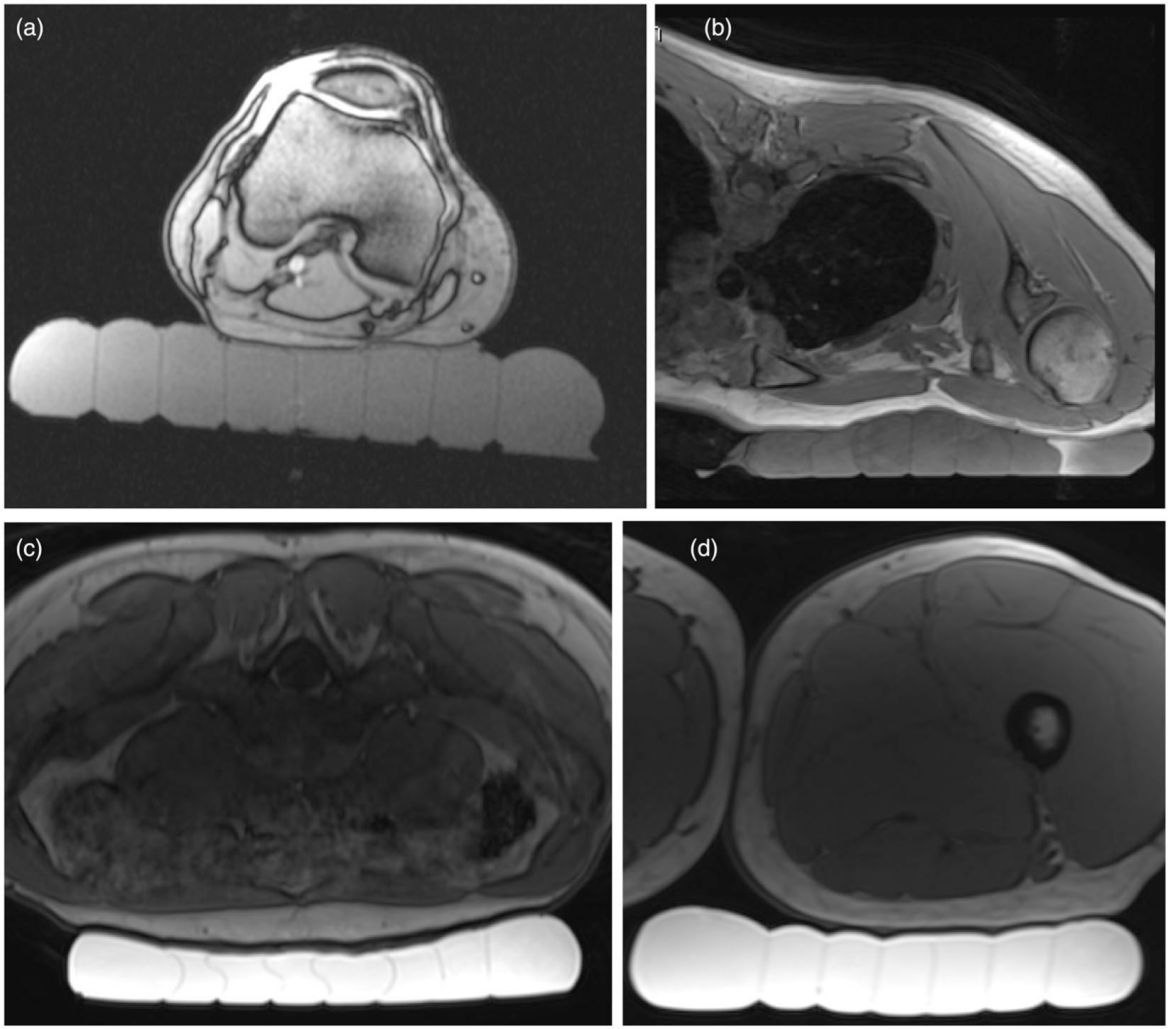
Demonstration of skin-cooling pad assembly conformability and acoustic coupling ability to four potential anatomical targets in a healthy volunteer. Axial T1w images are shown with the skin cooling pad coupled to (a) posterior knee, (b) inferior to the clavicle, (c) abdomen and (d) upper thigh. The subject is positioned prone in (b) and (c) and supine in (a) and (d). The skin-cooling pad assembly used in these tests had more interior channels that what is shown in Figure 1(c).

Skin-cooling pad assembly conformability and acoustic coupling quality are shown in Figure 7. Four potential anatomical targets were evaluated, specifically the lower extremity posterior to the knee, inferior to the clavicle, lower abdomen and lower extremity just below the buttock. In all cases, the pad was able to conform to the anatomy with no bubbles present between the pad and skin, indicating appropriate acoustic coupling would be achieved for acoustic transmission if degassed water was present above the level of the skin-cooling pad.

The energy applied for each sonication grid is shown in Table 1. While there were some user errors in energy application as indicated, the total energy applied per condition was not significantly different between the skin cooling and control conditions in any of the animals.

**Figure 8.**
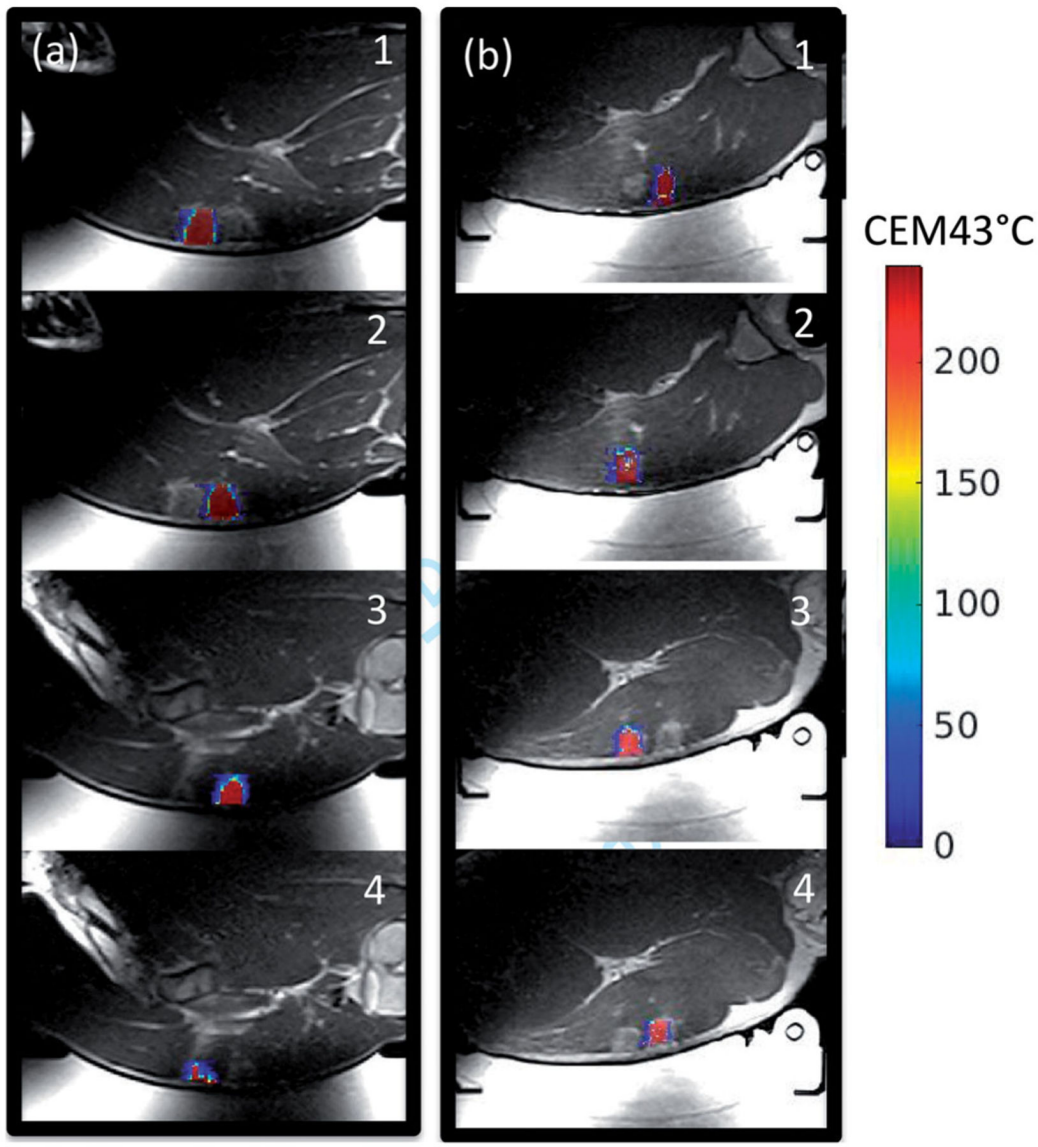
Comparison of control and skin-cooling conditions in animal 2. Cumulative thermal dose is overlaid on an axial T2-weighted image obtained at the end of the sonication period. Sonication matrices are labeled for both the (a) control and (b) skin-cooling conditions. The low thermal dose accumulation in the control condition, grid 4, was partially due to low image SNR from the MRI coil slipping from its original position.

The volumes of the cumulative thermal dose accumulated greater than 240 CEM43 °C is indicated for each sonication matrix as defined in Figure 5 and are listed in Table 2. While there is some variability between the thermal dose volumes achieved between the control and skin cooling conditions for each of the sonication matrices, there is not a significant difference with the mean ± one standard deviation being, 0.93 ± 0.55 cm^3^ and 1.08 ± 0.25 cm^3^, respectively. The thermal dose pattern overlaid on an axial T2-weighted image acquired after the sonications is shown for animal 3 in Figure 8. While there is some signal dropout in the T2-weighted images due to the RF coil placement and resulting sensitivity, the thermal dose measurements were obtained with a different sequence and were not impacted by this signal loss.

**Figure 9.**
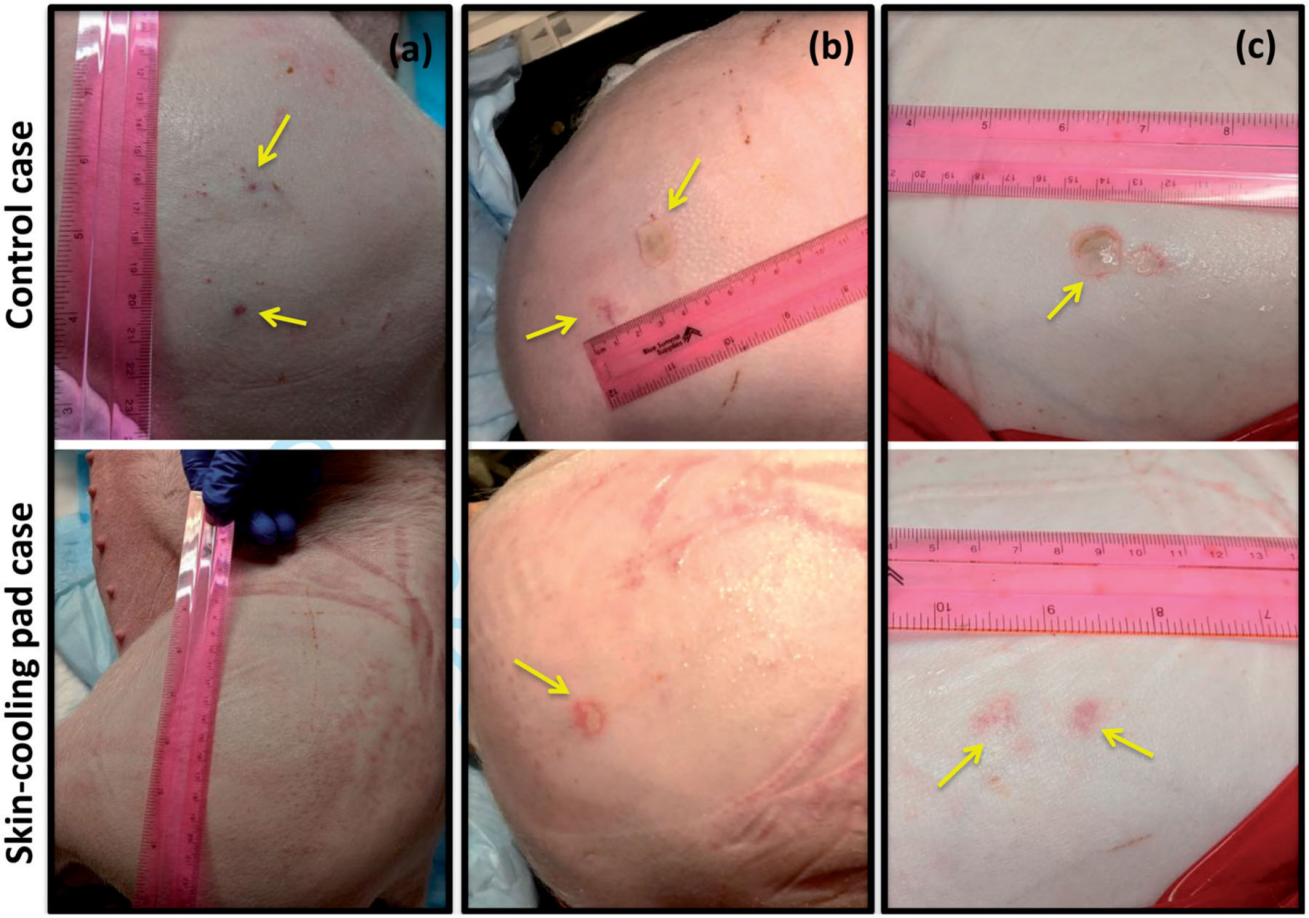
Gross assessment of the skin immediately post experiment. Any burns or other marks were noted for animals (a) 1, (b) 2 and (c) 3. The control condition side is shown in the top image with the skin-cooling condition shown in the bottom image. Burns or other marks are indicated with yellow arrows. The sonication matrices that resulted in burns or marks are indicated in Table 2.

The use of the skin-cooling pad allowed for thermal dose to be deposited close to the skin with a reduction of skin burn severity and size as seen in Figure 9. Sonication matrices that resulted in a burn or mark on the skin that later resolved are indicated in Table 2. In animal 1, ablation performed with the control condition did not result in skin burns, but acutely, three dark red marks were present on the skin, approximately 1 cm in size. These marks were the result of sonication matrices 2, 3, and 4. The marks had mostly resolved at the time of the picture, taken 160 min after the ablation was completed. The skin cooling condition allowed ablation immediate adjacent to the skin with no skin burns present at any time point. In animal 2, the control ablations left a skin burn approximately 2 cm in length with a transient mark that was assessed 176 min after the ablation, while the ablations on the skin-cooling side resulted in a skin burn of approximately 1 cm in length. The burns for the control and skin-cooling side occurred during sonication matrix 3. In animal 3, control ablations resulted in two skin burns of 2 and 1 cm in length during sonications 3 and 4, assessed 203 min after ablation completion, while the skin cooling ablations resulted in no skin burns, only discrete red marks during sonications 3 and 4 that resolved after approximately 15 min.

## Discussion

A conformable, MR-compatible, forced convective skin-cooling system for use with body target applications has been designed and evaluated. This system allows ablative acoustic exposures 5 mm away from the skin with reduced or eliminated skin burns when compared to a control condition of no skin-cooling pad assembly in place. The skin-cooling pad allows the near-field tissues to cool faster, potentially reducing overall treatment times, particularly in cases where many sonications are applied. The materials in contact with the patient and coolant fluid have no biocompatibility issues. The skin-cooling pad assembly can be used with flowing coolant during sonications. The data presented showed no degradation of the MR imaging during the planning, monitoring or assessment phases of the treatment. However, it should be noted that depending on the MR image acquisition geometry and phase encoding direction that there is potential for image artifacts in future use, and this should be considered when utilizing the skin-cooling pad assembly.

In all but one case, a mark or skin burn occurred when ablation occurred 5 mm from the skin. Due to the experimental protocol, any skin marks or burns resulting from ablations performed during the control condition were assessed approximately 3 h after ablation completed as the animal was still installed in the MRI scanner. Therefore, any acute markings were not photographically documented. In all animals, the skin burns were either eliminated or reduced by the presence of the skin-cooling pad. Sonication grid 3, which employed a 30 s sonication followed by a 30 s cooling period resulted in skin marks or burns in all but one case (animal 1, skin-cooling pad). The use of the skin-cooling pad either eliminated or reduced the size of the skin burn or mark, and allowed a larger volume of tissue to be ablated at the intended target. While the difference between the mean thermal dose volumes of the control and skin-cooling conditions was not significant, there was some variability in the achieved thermal dose volumes, as detailed in Table 2. The study was designed to apply equivalent cumulative energy and not equivalent thermal dose volumes. The thermal dose variability could be due to intra- and inter-animal response to the multiple sonications or changing acoustic and thermal properties as a function of temperature or tissue changes. Despite these differences in thermal dose volumes, the skin burns were reduced with the use of the skin-cooling pad assembly.

As with any aspect of MRgFUS treatments, acoustic coupling of the ultrasound beam to the patient is critical. The addition of the skin-cooling pad assembly to the acoustic window requires careful attention to ensure complete acoustic coupling is achieved with no air bubbles. In the pig experiments, successful acoustic coupling required the skin-cooling pad assembly to be covered with water, as shown in Figure 4(b). The conformable nature and feedback pressure control allow for the pad to be used at a variety of treatment sites under variable load conditions, as shown in Figure 7. This flexibility is required for treatment of desmoid tumors. The skin-cooling pad assembly was able to maintain pressure and conform to the desired anatomy with the pressure control system. The only pressure problem encounter occurred when the system operational pressure set-point was set below the baseline back-pressure and flow resistance of the assembly (including tubing lines, skin-cooling pad assembly and valves). It was found that a set-point value of approximately 115 mbar (at 1300 meters elevation) counteracted this potential problem and allowed pad coupling and conformability to be maintained throughout the study.

Hydrophone scans demonstrated a 5–12% reduction of peak pressure with use of the cooling pad. Since acoustic intensity is proportional to the square of the pressure magnitude, this equates to a 10–23% reduction in intensity. The measured acoustic impedance values of the fluid in the skin-cooling pad assembly and the coupling fluid used during the studies was 2.63%. Therefore, this loss of energy is due to a combination of absorption, reflection or refraction of the ultrasound beam. However, these acoustic effects did not appear to heat up the materials of the skin-cooling pad assembly, cause additional risk to the patient, or reduce the efficacy of the ablation at the intended target.

Utilization of the skin-cooling pad resulted in a more uniform thermal dose volume size when compared to the control condition as shown in Table 2. However, there is clear inter- and intra-animal variability in lesion volume when comparing equivalent experimental settings. In some cases, it is possible that the skin and subcutaneous tissues were damaged, simultaneously reducing the energy at the focal plan while potentially worsening skin damage. Because temperatures cannot be measured in the subcutaneous fat layer due to the limitations of the proton resonance frequency method, this effect could not be quantified. It is clear that a more uniform thermal dose volume size was achieved during the skin-cooling treatment condition, potentially due to the skin and subcutaneous tissue layers maintaining a low, uniform temperature during the sonications. This is of critical importance to long duration ablation procedures, as once the skin or fat layers are damaged or even heated to sublethal levels, attenuation values can increase significantly [41,42], not only increasing the chance of injury to those tissues, but also reducing the energy achieved at the treatment site. Additionally, utilizing the skin-cooling pad during long ablation procedures could potentially cut down the treatment time. The presented *in vivo* results demonstrate it is possible to reduce the inter-sonication delay time by a factor of two or more while simultaneously reducing or eliminating the possibility of skin burns. Therefore, a 90-sonication ablation treatment with a mean inter-sonication delay time of 45 s (assuming 1500 J sonications) [4] could reduce the total treatment inter-sonication delay time from approximately 68–34 min, providing a more efficient treatment environment.

## Conclusion

The conformable, forced convective skin-cooling system presented in this work has been demonstrated to reduce or eliminate skin burns during ablation of volumes as close as 5 mm from the skin. Sonications can be performed without turning off the flow with no impact on the MR temperature imaging precision.

This MR-compatible skin-cooling system is provided as an open source design. Component lists, design plans, assembly instructions, and user manual are provided to those who wish to construct their own device (https://github.com/fus-lab-uofu/SkinCoolingDevice). In principle, the skin-cooling pad assembly size could be customized to a specific MRgFUS system or application.

## Figures and Tables

**Table 1.** Energy applied at each of the sonication matrices as defined in Figure 4.

	Applied energy (kJ)			
	Control side		Skin-cooling side	
Animal	Energy for each sonication matrix [1–4]	Cumulative energy	Energy for each sonication matrix [1–4]	Cumulative energy

1	10.07, 7.88*, 9.37, 8.64	35.96	9.94, 8.79. 9.24, 8.45	36.42
2	12.10, 10.73, 8.53, 10.73	42.09	10.96, 10.08, 10.40, 10.73	42.17
3	10.96, 9.45, 10.96, 8.33	39.70	10.96, 9.30, 10.96, 9.76	40.98

The energy was adjusted to achieve an approximate temperature of 65 °C at each sonication, resulting in some applied power variance due to inter- and intra-animal variability. The energy for the 2nd sonication matrix on the control side of animal 1 (*) was low due to user error.

**Table 2.** Volume (cm^3^) that achieved a cumulative thermal dose of >240 CEM43 °C for each sonication matrix.

Animal Sonication matrix	1		2		3	
	Control	With skin-cooling pad	Control	With skin-cooling pad	Control	With skin-cooling pad

1	0.74	0.81	1.73 (M)	0.75	1.03	1.12
2	2.19 (M)	1.22	1.86	1.44	1.47	0.95
3	0.50 (M)	1.5	0.80 (B)	0.96 (B)	0.52 (B)	0.78 (M)
4	0.90 (M)	1.10	0.16*	1.07	0.48 (B)	1.27 (M)

Volumes are reported as a function of animal and whether achieved in a control or skin-cooling condition. Mean volume and one standard deviation for the control and skin-cooling pad condition sonications was 0.93 ± 0.55 and 1.08 ± 0.25 cm^3^, respectively. Sonications where temporary marks (M) or skin burns (B) occurred are indicated by a gray cell with an *M* or *B* designation, respectively.

(*) Low SNR was achieved during the thermometry due to the position of the MRI coil being shifted during the animal 2, sonication matrix 4 acquisition.

## References

[1] Tempany CM, Stewart EA, McDannold N, MR imaging-guided focused ultrasound surgery of uterine leiomyomas: a feasibility study. Radiology. 2003;226(3):897–905. 12616023 10.1148/radiol.2271020395

[2] Stewart EA, Gedroyc WM, Tempany CM, Focused ultrasound treatment of uterine fibroid tumors: safety and feasibility of a noninvasive thermoablative technique. Am J Obstet Gynecol. 2003;189(1):48–54. 12861137 10.1067/mob.2003.345

[3] Avedian RS, Bitton R, Gold G, Is MR-guided high-intensity focused ultrasound a feasible treatment modality for desmoid tumors? Clin Orthop Relat Res. 2016;474(3):697–704. 26040967 10.1007/s11999-015-4364-0 PMC4746191

[4] Ghanouni P, Dobrotwir A, Bazzocchi A, Magnetic resonance-guided focused ultrasound treatment of extra-abdominal desmoid tumors: a retrospective multicenter study. Eur Radiol. 2017;27(2):732–740. 27147222 10.1007/s00330-016-4376-5 PMC5097700

[5] Bucknor MD, Rieke V. MRgFUS for desmoid tumors within the thigh: early clinical experiences. J Ther Ultrasound. 2017;5:4. 28174660 10.1186/s40349-017-0081-3 PMC5290631

[6] Ghanouni P, Kishore S, Lungren MP, Treatment of low-flow vascular malformations of the extremities using MR-guided high intensity focused ultrasound: preliminary experience. J Vasc Interv Radiol. 2017;28(12):1739–1744. 29157478 10.1016/j.jvir.2017.06.002 PMC5726422

[7] Anzidei M, Napoli A, Sandolo F, Magnetic resonance-guided focused ultrasound ablation in abdominal moving organs: a feasibility study in selected cases of pancreatic and liver cancer. Cardiovasc Intervent Radiol. 2014;37(6):1611–1617. 24595660 10.1007/s00270-014-0861-x

[8] Escobar C, Munker R, Thomas JO, Update on desmoid tumors. Ann Oncol. 2012;23(3):562–569. 21859899 10.1093/annonc/mdr386

[9] Nuyttens JJ, Rust PF, Thomas CR Jr, Surgery versus radiation therapy for patients with aggressive fibromatosis or desmoid tumors: a comparative review of 22 articles. Cancer. 2000;88(7):1517–1523. 10738207

[10] Wang Y, Wang W, Tang J. Ultrasound-guided high intensity focused ultrasound treatment for extra-abdominal desmoid tumours: preliminary results. Int J Hyperthermia. 2011;27(7):648–653. 21797696 10.3109/02656736.2011.597047

[11] Li C, Zhang W, Zhang R, Superficial malignant tumors: noninvasive treatment with ultrasonographically guided high-intensity focused ultrasound. Cancer Biol Ther. 2009;8(24):2398–2405. 19923911 10.4161/cbt.8.24.10277

[12] Kovatcheva R, Guglielmina JN, Abehsera M, Ultrasound-guided high-intensity focused ultrasound treatment of breast fibroadenoma-a multicenter experience. J Ther Ultrasound. 2015;3(1):1. 25635224 10.1186/s40349-014-0022-3 PMC4310188

[13] Mougenot C, Kohler MO, Enholm J, Quantification of near-field heating during volumetric MR-HIFU ablation. Med Phys. 2011;38(1):272–282. 21361196 10.1118/1.3518083

[14] Payne A, Vyas U, Todd N, The effect of electronically steering a phased array ultrasound transducer on near-field tissue heating. Med Phys. 2011;38(9):4971–4981. 21978041 10.1118/1.3618729 PMC3166338

[15] Illing RO, Kennedy JE, Wu F, The safety and feasibility of extracorporeal high-intensity focused ultrasound (HIFU) for the treatment of liver and kidney tumours in a Western population. Br J Cancer. 2005;93(8):890–895. 16189519 10.1038/sj.bjc.6602803 PMC2361666

[16] Zhao Z, Wu F. Minimally-invasive thermal ablation of early-stage breast cancer: a systemic review. Eur J Surg Oncol. 2010;36(12):1149–1155. 20889281 10.1016/j.ejso.2010.09.012

[17] Jung SE, Cho SH, Jang JH, High-intensity focused ultrasound ablation in hepatic and pancreatic cancer: complications. Abdom Imaging. 2011;36(2):185–195. 20512487 10.1007/s00261-010-9628-2

[18] Leon-Villapalos J, Kaniorou-Larai M, Dziewulski P. Full thickness abdominal burn following magnetic resonance guided focused ultrasound therapy. Burns. 2005;31(8):1054–1055. 15970389 10.1016/j.burns.2005.04.019

[19] Gianfelice D, Khiat A, Boulanger Y, Feasibility of magnetic resonance imaging-guided focused ultrasound surgery as an adjunct to tamoxifen therapy in high-risk surgical patients with breast carcinoma. J Vasc Interv Radiol. 2003;14(10):1275–1282. 14551274 10.1097/01.rvi.0000092900.73329.a2

[20] Kennedy JE, Wu F, ter Haar GR, High-intensity focused ultrasound for the treatment of liver tumours. Ultrasonics. 2004;42(1–9):931–935. 15047409 10.1016/j.ultras.2004.01.089

[21] Furusawa H, Namba K, Nakahara H, The evolving non-surgical ablation of breast cancer: MR guided focused ultrasound (MRgFUS). Breast Cancer. 2007;14(1):55–58. 17244995 10.2325/jbcs.14.55

[22] Damianou C, Hynynen K. Focal spacing and near-field heating during pulsed high temperature ultrasound therapy. Ultrasound Med Biol. 1993;19(9):777–787. 8134978 10.1016/0301-5629(93)90094-5

[23] Fan X, Hynynen K. Ultrasound surgery using multiple sonications–treatment time considerations. Ultrasound Med Biol. 1996;22(4):471–482. 8795174 10.1016/0301-5629(96)00026-9

[24] Rueff LE, Raman SS. Clinical and technical aspects of MR-guided high intensity focused ultrasound for treatment of symptomatic uterine fibroids. Semin Intervent Radiol. 2013;30(4):347–353. 24436561 10.1055/s-0033-1359728 PMC3835439

[25] Von Ungern-Sternberg BS, Habre W. Pediatric anesthesia – potential risks and their assessment: part I. Paediatr Anaesth. 2007;17(3):206–215. 17263734 10.1111/j.1460-9592.2006.02097.x

[26] Kim YS, Bae DS, Park MJ, Techniques to expand patient selection for MRI-guided high-intensity focused ultrasound ablation of uterine fibroids. AJR Am J Roentgenol. 2014;202(2):443–451. 24450690 10.2214/AJR.13.10753

[27] Kim YS, Keserci B, Partanen A, Volumetric MR-HIFU ablation of uterine fibroids: role of treatment cell size in the improvement of energy efficiency. Eur J Radiol. 2012;81(11):3652–3659. 21959213 10.1016/j.ejrad.2011.09.005

[28] Kohler MO, Mougenot C, Quesson B, Volumetric HIFU ablation under 3D guidance of rapid MRI thermometry. Med Phys. 2009;36(8):3521–3535. 19746786 10.1118/1.3152112

[29] Baron P, Ries M, Deckers R, In vivo T2 -based MR thermometry in adipose tissue layers for high-intensity focused ultrasound near-field monitoring. Magn Reson Med. 2014;72(4):1057–1064. 24259459 10.1002/mrm.25025

[30] Ikink ME, van Breugel JM, Schubert G, Volumetric MR-guided high-intensity focused ultrasound with direct skin cooling for the treatment of symptomatic uterine fibroids: proof-of-concept study. Biomed Res Int. 2015;2015:684250. 26413538 10.1155/2015/684250 PMC4568047

[31] Ghanouni P, Pauly KB, Elias WJ, Transcranial MRI-guided focused ultrasound: a review of the technologic and neurologic applications. AJR Am J Roentgenol. 2015;205(1):150–159. 26102394 10.2214/AJR.14.13632 PMC4687492

[32] Joo B, Park M-S, Lee SH, Pain palliation in patients with bone metastases using magnetic resonance-guided focused ultrasound with conformal bone system: a preliminary report. Yonsei Med J. 2015;56(2):503–509. 25684002 10.3349/ymj.2015.56.2.503 PMC4329365

[33] Lee HJ, Lee MH, Lee SG, Evaluation of a novel device, high-intensity focused ultrasound with a contact cooling for subcutaneous fat reduction. Lasers Surg Med. 2016;48(9):878–886. 27551954 10.1002/lsm.22576

[34] Fruehauf JH, Back W, Eiermann A, High-intensity focused ultrasound for the targeted destruction of uterine tissues: experiences from a pilot study using a mobile HIFU unit. Arch Gynecol Obstet. 2008;277(2):143–150. 17823809 10.1007/s00404-007-0435-0

[35] Hiraoka M, Jo S, Dodo Y, Clinical results of radiofrequency hyperthermia combined with radiation in the treatment of radioresistant cancers. Cancer. 1984;54(12):2898–2904. 6498766 10.1002/1097-0142(19841215)54:12<2898::aid-cncr2820541214>3.0.co;2-b

[36] Zenzie HH, Altshuler GB, Smirnov MZ, Evaluation of cooling methods for laser dermatology. Lasers Surg Med. 2000;26(2):130–144. 10685086 10.1002/(sici)1096-9101(2000)26:2<130::aid-lsm4>3.0.co;2-j

[37] Wilson TE, Cui J, Zhang R, Skin cooling maintains cerebral blood flow velocity and orthostatic tolerance during tilting in heated humans. J Appl Physiol. 2002;93(1):85–91. 12070190 10.1152/japplphysiol.01043.2001

[38] Goodman JW. Introduction to Fourier optics. 3rd ed. Englewood (CO): Roberts & Company Publishers; 2004.

[39] Farrer AI, Odeen H, de Bever J, Characterization and evaluation of tissue-mimicking gelatin phantoms for use with MRgFUS. J Ther Ultrasound. 2015;3:9. 26146557 10.1186/s40349-015-0030-y PMC4490606

[40] Sapareto SA, Dewey WC. Thermal dose determination in cancer therapy. Int J Radiat Oncol Biol Phys. 1984;10(6):787–800. 6547421 10.1016/0360-3016(84)90379-1

[41] Clarke RL, Bush NL, Ter Haar GR. The changes in acoustic attenuation due to *in vitro* heating. Ultrasound Med Biol. 2003;29(1):127–135. 12604124 10.1016/s0301-5629(02)00693-2

[42] Ghoshal G, Luchies AC, Blue JP, Temperature dependent ultrasonic characterization of biological media. J Acoust Soc Am. 2011;130(4):2203–2211. 21973375 10.1121/1.3626162 PMC3206913

